# Prevalence and Associations, by Age Group, of IPV Among AGYW in Rural South Africa

**DOI:** 10.1177/2158244019830016

**Published:** 2019-02-18

**Authors:** Amanda Selin, Stephanie M. DeLong, Aimée Julien, Catherine MacPhail, Rhian Twine, James P. Hughes, Yaw Agyei, Erica L. Hamilton, Kathleen Kahn, Audrey Pettifor

**Affiliations:** 1 The University of North Carolina at Chapel Hill, Chapel Hill, NC, USA; 2 University of Wollongong, New South Wales, Australia; 3 Medical Research Council/Wits University Rural Public Health and Health Transitions Research Unit (Agincourt), School of Public Health, Faculty of Health Sciences, University of the Witwatersrand, Johannesburg, South Africa; 4 Wits Reproductive Health and HIV Institute, University of the Witwatersrand, Johannesburg, South Africa; 5 University of Washington, Seattle, WA, USA; 6 Johns Hopkins University School of Medicine, Baltimore, MD, USA; 7 Science Facilitation Department, FHI 360, Durham, NC, USA

**Keywords:** Africa, data processing and interpretation, research methods, social sciences

## Abstract

The prevalence of intimate partner violence (IPV) is alarmingly high among South African adolescent girls and young women (AGYW). Limited data exist exploring how IPV prevalence and its risk factors differ by age. Study data were from the baseline visit of HPTN 068, a randomized controlled trial (RCT) conducted from 2011 to 2015 in Mpumalanga, South Africa. A cohort of 2,533 AGYW, aged 13 years to 20 years, answered survey questions on demographics and behaviors, including their experiences of physical and sexual violence ever and in the past 12 months. We calculated the prevalence of IPV and related risk factors, as well as prevalence ratios with 95% confidence intervals, stratified by age. Nearly one quarter (19.5%, 95% CI = [18.0, 21.2]) of AGYW experienced any IPV ever (physical or sexual) by a partner. The prevalence of any IPV ever among AGYW aged 13 years to 14 years, 15 years to 16 years, and 17 years to 20 years was 10.8%, 17.7%, and 32.1%, respectively. Key variables significantly associated with any IPV ever across all age groups included borrowing money from someone outside the home in the past 12 months, ever having had vaginal sex, ever having had anal sex, and consuming any alcohol. Few statistically significant associations were unique to specific age groups. The history of IPV among the youngest AGYW is a critical finding and should be a focus of prevention efforts.

## Background

### Global Intimate Partner Violence (IPV)

Women’s experience of IPV is a global concern, and intervention efforts to decrease the burden of IPV are vital. A 2013 World Health Organization (WHO) report indicated that globally, close to one third of women who reported ever having been in a relationship experienced IPV, and nearly 30% of women in southern sub-Saharan Africa report experiencing it (García-Moreno et al., 2013). In South Africa, approximately one fifth of adult women report ever experiencing violence by a partner (Statistics South Africa, 2017).

### South African Adolescent Girls and Young Women (AGYW) and IPV

IPV prevalence estimates are also alarmingly high among South African AGYW. A multicountry study published in 2014 found that among 15-year to 19-year-old adolescent young women in Johannesburg, prevalence in the past year of physical IPV was 30.9% and sexual IPV was 18.3% (Decker et al., 2014). Similarly, a 2015 study in a peri-urban area of South Africa found that 16-year to 24-year-old AGYW who reported multiple partnerships also reported a high burden of IPV; 80% reported physical IPV and 67% reported sexual IPV in the past 12 months (Zembe, Townsend, Thorson, Silberschmidt, & Ekstrom, 2015). Furthermore, a study in urban South Africa found that among Grade 8 AGYW, lifetime experience of physical and sexual IPV was 24.1% and 13.8%, respectively; and 30.9% of AGYW reported either physical or sexual IPV. The prevalence was also delineated by age group (12–13 years vs. 14–19 years), and the prevalence of sexual or physical IPV was higher among the older age group (46.8% vs. 53.2%; Shamu et al., 2016). A Cape Town study of Grade 8 AGYW found that 39% reported physical IPV by a partner in the past 3 months (Russell et al., 2014).

### Risk Factors and IPV

Several factors are associated with IPV in South Africa, including violence as the acceptable social norm (Jewkes, 2002), childhood experiences of abuse (Shamu et al., 2016), alcohol and substance use (Decker et al., 2014; Shamu et al., 2016), multiple partnerships (Decker et al., 2014), transactional sex (Decker et al., 2014; Zembe et al., 2015), lack of condom use (Decker et al., 2014), and mental health problems, such as depression (Decker et al., 2014).

### Adolescent Development

Although there are several prevalence studies with reported risk factors for IPV among South African AGYW, most studies aggregate ages and do not provide information separated into the three stages of adolescence—early adolescence, middle adolescence, and later adolescence entering young adulthood. Key life events are transpiring at different ages (e.g., beginning of romantic and sexual relationships in middle adolescence), which may influence external exposures as well as the behaviors of the AGYW. Early adolescence is marked by the start of puberty (Rosenfield, Lipton, & Drum, 2009), budding sexual attraction, the preparation for or entry into secondary school, and wanting to fit in closely with peers (Teipel, 2003a). This may be the time when the adolescent is introduced to her first partnership. In middle adolescence, AGYW often begin to have sex and conflict with parental figures may escalate as they attempt to establish their autonomy (e.g., spending more of their free time with partners). During this period, peer acceptance on partner choice and emulating the behaviors they perceive their peers to be engaging in is important (Teipel, 2003c). In older adolescence, AGYW are preparing for the adult stage of life. This may include graduation from secondary school and/or preparing for tertiary education, a full-time job, and raising a family. At this stage, much of the physical growth from the earlier stages of adolescence begins slowing down. Another key component to this developmental stage is the transition from predominantly peer-oriented relationships to more intimate and serious relationships. During this stage, the AGYW are beginning to solidify characteristics they are looking for in a partner (Teipel, 2003b).

In the early 2000s, Arnett coined the term “emerging adulthood,” conceptualizing adolescence as a period that could extend through the 20s (Arnett, 2000). Many resource-rich settings can support an extended period of adolescent development and exploration, but AGYW living in regions with particular economic and social expectations may take on more traditional adult roles, such as parenting, at a younger age, limiting their opportunities (Sawyer, Azzopardi, Wickremarathne, & Patton, 2018). A rural Mpumalanga, South African study, found adult women identified puberty, socializing with the opposite sex, having a child (regardless of marital status), and behaving in a socially respectable and feminine manner as important components to a successful transition to womanhood (Sennott & Mojola, 2017). Biologically, changes in social-affective processing during adolescence may position the AGYW to be more adaptive to changing contexts, therefore, more willing to consider and enact new ideas and behaviors when presented with them (Crone & Dahl, 2012). This transitional time period could mark an uptick in risky behaviors associated with IPV, but just as important, both adolescent women and men may show an increased preparedness, motivation and flexibility to engage in interventions that help prevent IPV, if presented with them.

### IPV Prevalence and Stages of Adolescence

In this paper, we provide prevalence estimates of IPV and explore potentially related factors, such as individual, partner, and economic characteristics, disaggregated by age group (13–14, 15–16, 17–20; all in years) among AGYW in rural Mpumalanga Province, South Africa. Our cohort ranged in age from 13 years to 20 years, with a median age of 15 years. The defined age range for each developmental stage differs across studies (Curtis, 2015), which can make cross-study comparisons challenging. The disaggregation we chose closely matches the typical ranges for the three stages of adolescence, while considering the data available in our study. We contribute to the existing body of evidence by highlighting these differences by age strata.

## Method

### Study Overview

This secondary analysis uses baseline data from HPTN 068, an RCT conducted in Mpumalanga, South Africa. The HIV Prevention Trials Network (HPTN) conducts global clinical trials focused on HIV prevention. The purpose of HPTN 068 was to assess if receiving cash transfers, conditional on school attendance, reduced HIV incidence in South African AGYW. Each AGYW randomized to the intervention arm and her parent/legal guardian received approximately US$30 per month, in total, if she successfully attended 80% of school the month prior. Baseline study data were collected from March 2011 through December 2012 (Pettifor, MacPhail, Selin, et al., 2016). This pre-intervention data set provided a unique opportunity to analyze IPV among a large sample of South African AGYW, including young AGYW, disaggregated by age.

### Enrollment

The Agincourt Health and Socio-Demographic Surveillance System (AHDSS) follows the local population through an annual census to monitor health and social factors. This census served as the platform for recruitment of the study sample (Kahn et al., 2012). The AHDSS has a strong relationship with the communities engaged in the research, and prior to study start, each study is formally introduced to the participating communities. The community also receives summarized study results upon analysis completion.

To be enrolled in the study, participants needed to be female and between 13 years and 20 years of age at baseline, planning to reside in the AHDSS catchment area for the next 3 years, a student in Grades 8 to 11 at one of the local 26 public high schools, able to consent or assent to participate in the study, be able to read, have either a bank account or the proper paperwork to open a bank account to receive the cash transfer (if randomized to the intervention arm), and not be pregnant or married at baseline. An HIV positive status at baseline was not an exclusion criterion for study participation. A parent/legal guardian also needed to consent to the AGYW’s participation (if she was below 18 years), as well as consent to her or his own participation. In addition, the parent/legal guardian needed to also either have a bank account, have proper paperwork to open a bank account, or be able to identify someone who could receive the money. There were 2,533 AGYW and their parents/legal guardians enrolled in the study, 25% of the total number of families screened (Pettifor, MacPhail, Hughes, et al., 2016); all participants enrolled provided informed consent and assent.

### Data Collection and Baseline Measures

Data for this analysis were acquired from two surveys and from laboratory data. The surveys included a baseline household survey on family economic assets conducted with the parent/guardian of the AGYW and then the AGYW baseline survey, from which most of the data for the analysis originates.

#### Baseline household survey.

Parents/legal guardians of the AGYW completed a fieldworker led computer survey at home using Computer-Assisted Personal Interview (CAPI) software. The household survey included a roster of characteristics of those living in the home, agricultural and water resources, durable assets, and monetary transfers in and out of the home.

#### Young women’s survey and laboratory testing.

To maintain privacy for sensitive questions, most of the data collection process involved use of Audio Computer-Assisted Self-Interview (ACASI), which allowed young women to read and hear the questions through headphones and complete the survey on their own, potentially minimizing response bias. Survey questions included IPV experience, alcohol use, views toward gender norms, number of sexual partners, coital debut, and number of self-reported sex acts in the past 3 months, among others. A majority of the predictor variables were analyzed with a dichotomous response of yes/no. Age at sexual debut (15 years and younger vs. older than 15 years), number of lifetime partners (two or more vs. zero or one), and orphan status (one or both parents deceased vs. none) are examples of the different variable categorizations. The gender norms scale focused on sexual relationship domains, household chores and daily life, and reproductive health and disease prevention. There were 13 questions asked, ranging from, “It is the man who decides what type of sex to have” to “A woman should obey her husband in all things.” AGYW could respond with “agree a lot,” “somewhat agree,” or “do not agree.” Final scores were calculated, with the most equitable gender norms being a score of 13 and the least being a score of 0. Pretest counseling for HIV occurred after the survey, followed by HIV testing, posttest counseling, and finally randomization for the overall trial. Participants received HIV rapid test results on the same day the testing was performed.

#### Definition and measurement of IPV in the young women’s baseline survey.

IPV was defined as physical and/or sexual violence occurring between an AGYW and a partner, with partnership defined as a “current boyfriend or partner or any other partner in [a young woman’s] past.” There were a total of eight IPV questions asked, designed by the WHO (García-Moreno, Jansen, Ellsberg, Heise, & Watt, 2005), including six physical violence questions and two sexual violence questions. The severity of the physical violence ranged from “Has a partner ever slapped you or threw something at you that could hurt?” to “Has a partner ever threatened to use or used a gun, knife, or other weapon against you?”

Both physical and sexual violence questions assessed whether that form of violence had “ever” happened, and if yes, whether it had happened “in the last 12 months.” Physical violence questions specifically mentioned a partner as the perpetrator. Sexual violence questions asked about several kinds of perpetrators: a boyfriend/partner, a family member, or someone else outside the family (not including a boyfriend or partner). For this analysis, we only focused on sexual violence experienced by a partner.

### Study Response to Reports of Violence

During the post-test HIV counseling session, the counselor followed up with AGYW who reported violence in their surveys and provided them with rights and safety planning, which included information on health and sexual rights, strategies to stay safe, and how to handle situations that are physically or sexually unsafe. Following South African Health Department Guidelines, all reports of violence by AGYW below the age of 18 years were referred to a local social worker. AGYW and their parents/legal guardians were made aware of this procedure during the time of assent/consent, and again for the AGYW at time of survey completion. Social workers then contacted the AGYW to determine next steps, and the study team facilitated initial meetings between the AGYW and the social workers after triaging cases. AGYW 18 years and above who reported violence and wanted support from the social workers were also referred.

### Analysis

Data were analyzed in SAS v9.3. Prevalence and prevalence ratios with 95% confidence intervals were calculated using log binomial regression. Data were coded in the following manner: any IPV (responding yes to at least one physical or sexual IPV question), any physical IPV (answering yes to one or more physical IPV questions), any sexual IPV (answering yes to one or more sexual IPV questions), and both IPV (responding yes to at least one physical IPV question and yes to at least one sexual IPV question). If data on one or more questions within each type (i.e., any IPV) were missing, the constructed variable value was set to missing. Following this approach may have led to a slight underestimate of IPV reported.

## Results

### Study Population Characteristics

The 2,533 participants ranged in age from 13 years to 20 years, with a median age of 15 years. The 15- to 16-year olds were the largest age group, at 42.4%. Over one third of the participants reported being worried about having enough food for themselves or their family in the past 12 months, and 25% currently had savings for the future. Over one fifth needed to borrow money from outside the home during the past 12 months, and over one quarter had at least one parent who had died.

Just over one quarter (26.6%) of the sample reported ever having had vaginal sex, with a median sexual debut age of 16 years. As expected, a much lower number reported ever having had anal sex (4.8%). Among those who reported ever having had vaginal or anal sex, the median number of lifetime sexual partners was one. Among those who reported ever having had vaginal or anal sex, the majority (94.3%) also reported having had one or more partners in the past 12 months, indicating that while partnership number was low, it was recent. Over one quarter reported unprotected sex in the past 3 months and a large majority of participants who reported ever having had sex indicated that one or more of their sexual partners was a main partner/boyfriend (74%). Moreover, 14.1% of AGYW reported having had transactional sex—defined as sex in exchange for money or goods.

Approximately 9% of the young women in the total sample reported ever being pregnant, 25.8% reported currently using birth control, 3.2% were found HIV+ at baseline, and 4.7% were found HSV-2+ at baseline. Low substance abuse was also reported in our sample, with only 8.9% reporting any alcohol consumption (whether frequent or infrequent use), and 4.8% reporting ever having used drugs (Table 1).

### Prevalence of Experiencing IPV

**Figure 1.**
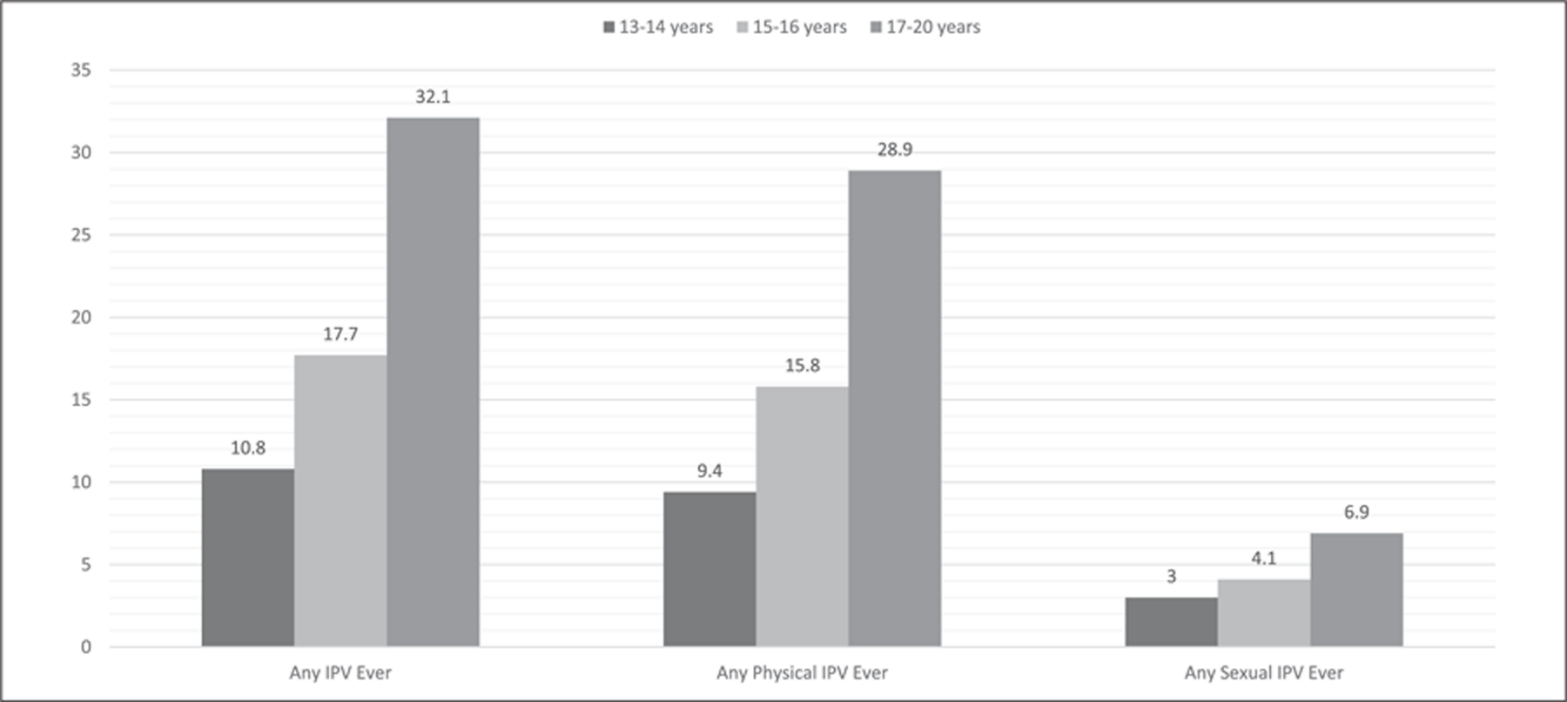
IPV prevalence by type and age group at HPTN 068 baseline. *Note.* IPV = intimate partner violence; HPTN = HIV Prevention Trials Network.

Close to 20% of our study population (19.5%, 95% CI = [18.0, 21.2]) ever experienced any IPV (physical or sexual) by a partner (Table 2). In addition, prevalence of experiencing any IPV increases with age (Figure 1). The prevalence of any IPV ever among AGYW aged 13 years to 14 years, 15 years to 16 years, and 17 years to 20 years was 10.8%, 17.7%, and 32.1%, respectively. The prevalence of any physical IPV and any sexual IPV was highest among the 17- to 20-year olds at 28.9% and 6.9%, respectively.

### Factors Associated With IPV

The prevalence of any IPV ever among AGYW with exposed status for selected characteristics is presented in Table 3. AGYW of all ages who had experienced IPV had a higher prevalence of worrying about food for self or family in the past 12 months, borrowing money outside the home in the past 12 months, having had vaginal sex, having had anal sex, and having consumed any alcohol (Table 4).

In addition, among both the 17- to 20-year olds and 15- to 16-year olds, ever being pregnant, currently using birth control, and having lower gender equitable norm scores were associated with experiencing any IPV ever (Table 4).

Furthermore, among the 17- to 20-year olds alone, currently having savings for the future, having unprotected sex with a partner in the past 3 months, having had transactional sex (among those who had ever had vaginal or anal sex), and ever using drugs were all factors significantly associated with experiencing IPV. Among the 15- to 16-year olds alone, currently living with a sex partner and having a casual sex partner had a significant association with IPV (Table 4).

## Discussion

In this study, among AGYW in rural South Africa, we found that the prevalence of sexual or physical IPV experienced was close to 20%. Given that 15 was the mean age of the sample and 26.6% reported ever having had vaginal sex, this is very high. Our study is the only study, of which we are aware, that describes the prevalence and associated factors of IPV among South African AGYW, disaggregated by age.

Physical violence is very common among AGYW who have had sex, whereas sexual violence from a partner is less common. Milder types of physical IPV are more prevalent compared with more severe types, reported both in their lifetime as well as in the past 12 months. Physical IPV (10.7%) was reported among AGYW who said they had never had sex (data not shown). This is not unexpected given AGYW may form partnerships not involving sex, especially during early adolescence, and thus, IPV interventions should be tailored accordingly. In addition, AGYW may have chosen not to report their sexual history in the survey.

Although our data suggest that age matters when having ever experienced any IPV, we did not see a great difference, by age, in risk factors for IPV. Rather, a common set of risk factors emerged for experiencing any IPV. This included borrowing money from someone outside the home in the past 12 months (a marker of experiencing poverty or insufficient resources for perceived needs), ever having had vaginal sex, ever having had anal sex, and consuming any alcohol.

In our study, borrowing money was associated with increased reporting of IPV among all age groups. Research in Kenya showed that working versus not working, among married female adolescents who did not have any savings, was associated with increased IPV, but male partner trust of the adolescent with money was associated with reduced IPV (Muthengi, Gitau, & Austrian, 2016). This supports the role of traditional gender roles (women not working) as well as male-centered decision making (his level of trust) in the experience of IPV. Among the 17- to 20-year olds in our study, having personal assets (i.e., personal savings for the future) was associated with IPV. A meta-analysis of global demographic and health surveys did not find a clear directional relationship between asset ownership and IPV (Peterman, Pereira, Bleck, Palermo, & Yount, 2017).

A minority of our sample reported ever having vaginal sex, ever having anal sex, and two or more lifetime partners. National data on South African adolescent sexual behavior (Shisana et al., 2014) supports this. Reporting ever having vaginal sex and ever having anal sex was associated with IPV among all age groups. Engaging in sex, particularly with multiple partners, introduces, as well as reinforces, social norms within relationships, that could be beneficial (e.g., trust and companionship), but also harmful (e.g., low support of gender equity, sexual expectations and roles). Consumption of alcohol was also associated with IPV among all age groups, and is established in the literature (Davis, Rotheram-Borus, Weichle, Rezai, & Tomlinson, 2017). Consuming alcohol may encourage attendance to alcohol venues (e.g., shebeens), which has been found to be associated with risky behavior (Rosenberg et al., 2015).

Transactional sex and drug use was associated with IPV among the 17- to 20-year olds. The association between transactional sex and IPV is known (Dunkle et al., 2007), but many research findings on the relationship between drug use and IPV report on drug use by the perpetrator rather than the victim. Among the middle and oldest adolescent age groups (ages 15–16, 17–20; in years), using birth control, ever being pregnant, and low support for gender equity were associated with IPV, as noted in the literature, respectively (Alio, Daley, Nana, Duan, & Salihu, 2009; Decker et al., 2014; Mpondo, Ruiter, van den Borne, & Reddy, 2016). Median sexual debut age is 16 years; therefore, the use of birth control and pregnancy could be reflecting sexual activity as the AGYW enter middle adolescence. Among AGYW aged 15 to 16 years, currently living with one or more (up to three) recent sex partners was associated with IPV, suggesting that cohabiting with a partner could place AGYW at an increased risk of IPV.

Our analyses show a low prevalence of IPV risk factors (e.g., ever sex, alcohol use, ever pregnant), but AGYW who experience IPV are more likely to report them. A comprehensive intervention package that addresses multiple risk factors of IPV is needed. To have lasting impact, interventions should be rooted in theories of behavior change and address the social norms that encourage male perpetration of violence (Jewkes, Flood, & Lang, 2015). A review of interventions targeting the reduction of IPV perpetration found that interventions were successful when they were delivered in multiple settings (e.g., both community and with parents), included adults who played a significant role in the life of the adolescent, had a longer duration, and addressed multiple types of violence (De Koker, Mathews, Zuch, Bastien, & Mason-Jones, 2014). As our analysis has shown, physical (both minor and major types) and sexual IPV are experienced by AGYW across the adolescent age spectrum.

Including parental figures in IPV prevention could help both adults and adolescents navigate some of the challenges faced during this time. The innovative U.S.-based intervention, *Families for Safe Dates*, positively impacted factors related to parental engagement around dating violence issues, showed lower acceptance of dating violence by the adolescent, and a smaller number of adolescents reported dating abuse onset, compared with the control group (Foshee et al., 2012). Exposure to familial violence as a child is a known risk factor for perpetration of IPV as an adult (Abrahams & Jewkes, 2005); therefore, interventions that also address exposure to violence at home may be protective. AGYW just entering adolescence are already experiencing alarming rates of IPV, and interventions with a particular focus on pre- and early adolescence may be beneficial. A recent school-based intervention with early and middle-aged adolescent boys and girls in Kenya demonstrated a reduction in IPV risk among the girls, at the school level. The program utilized a multicomponent curriculum that focused on promoting gender equitable behavior with the boys and empowerment, gender relations, and self-defense training for the girls. There was also a significant increase in reported self-efficacy among the girls, which could have longer lasting positive effects as they build more relationships throughout adolescence and adulthood (Baiocchi et al., 2017).

### Limitations

We acknowledge some limitations regarding our study. The data presented in this analysis are cross-sectional; therefore, we cannot make causal conclusions. Our IPV measures do not allow us to pinpoint exact time frames for when the violence occurred in the lifetime of the AGYW. However, we did use a common WHO multicountry study survey tool with standard time periods of measurement (ever and the past 12 months) in our study to best ascertain IPV experienced. Underreporting of IPV is possible and has been noted elsewhere (Palermo, Bleck, & Peterman, 2014). In our study, this could be due to both social desirability and knowing that, if below 18 years, participant-confirmed reports of IPV would be reported to a social worker. However, we attempted to minimize underreporting to the extent possible by the use of the ACASI survey tool in private. To note, some of the AGYW aged 18 years and older were interested in seeking services, even though referral was not mandatory.

### Conclusion

The key findings in this study are important as we look to the future of IPV prevention. Intervention efforts should address the experience of IPV among AGYW of all ages, but of note, among younger AGYW in their first relationships. Although our results showed some characteristics were associated with IPV across all age groups, a few, such as drug use, were found to only be significantly associated with certain age groups (in this case, within the oldest AGYW). More research, including longitudinal analysis on IPV among South African AGYW, disaggregated by age, would further elucidate the risk factors and age-specific findings of the current study. Researchers should consider that AGYW as young as 13 years, prior to the median age of sexual debut, are experiencing IPV. Partnering with adolescents and executing theory- and evidence-driven IPV prevention efforts must be considered a top priority.

## Figures and Tables

**Table 1.** Characteristics of South African AGYW Participating in HPTN 068 Baseline, March 2011-December 2012 (*n* = 2,533).

Characteristics	Yes, *n* (%)	Median (IQR)
Age in years (*n* = 2,533)		15 (14–17)
17–20^a^	685 (27.0)	
15–16	1,075 (42.4)	
13–14	773 (30.5)	
Orphan status (*n* = 2,406)		
One or both parents dead	683 (28.4)	
Past 12 months, worried about food for self or family (*n* = 2,511)	862 (34.3)	
Past 12 months, borrowed money from someone outside the home (*n* = 2,511)	563 (22.4)	
Currently has savings for the future (cash, assets; *n* = 2,520)	630 (25.0)	
Assets (number of durable goods in the home; *n* = 2,528)^b^		13 (9–18)
Assets (AGYW with ≤ 13 durable goods in the home)	1,317 (52.10)	
Ever had vaginal sex (*n* = 2,523)	672 (26.6)	
Age at vaginal sexual debut (among those who reported ever having vaginal sex; *n* = 631)^c^		16 (15–16)
15 years or younger	290 (46.0)	
Ever had anal sex (*n* = 2,528)	120 (4.8)	
Number of lifetime sexual partners (*n* = 676)^d^		1 (1–2)
Two or more	323 (47.8)	
One	353 (52.2)	
Number of sexual partners in past 12 months (*n* = 665)^d^		
One or more	627 (94.3)	
Zero	38 (5.7)	
Had unprotected sex with a partner in the past 3 months (*n* = 677)^d^	204 (30.1)	
One or more of the three most recent sexual partners is/was a main partner/boyfriend (*n* = 678)^d^	499 (73.6)	
One or more of the three most recent sexual partners is/was a regular, casual sex partner (*n* = 678)^d^	200 (29.5)	
One or more of the three most recent sexual partners is/was a nonregular casual sex partner (*n* = 678)^d^	94 (13.9)	
One or more of the three most recent sexual partners is/was a sex work client (*n* = 678)^d^	16 (2.4)	
Currently lives with one or more (up to three) of the three most recent sexual partners (*n* = 679)^d^	84 (12.4)	
Had transactional sex (sex because $ or gifts given; *n* = 689)^d^	97 (14.1)	
HIV+ (*n* = 2,529)	81 (3.2)	
HSV-2+ (*n* = 2,529)	120 (4.7)	
Had an older partner (≥5 years, sexual or nonsexual; *n* = 985)	153 (15.5)	
Gender equitable norms (GEMS; *n* = 2,530)^e^		4 (2–6)
Score of 0–4	1,447 (57.2)	
Score >4, up to 13	1,083 (42.8)	
Ever pregnant (*n* = 2,502)	223 (8.9)	
Currently using birth control (*n* = 2,520)	651 (25.8)	
Consumes any alcohol (*n* = 2,526)	225 (8.9)	
Ever used drugs (*n* = 2,527)	120 (4.8)	

*Note.* AGYW = adolescent girls and young women; HPTN = HIV Prevention Trials Network; HSV = herpes simplex virus; GEMS = gender equitable measurement scale; IQR = interquartile range.

a One participant turned 21 years on her date of randomization.

b Durable assets include items such as radios, televisions, cell phones, and so on.

c Vaginal sexual debut of ≤5 was considered unlikely and not included (*n* = 31).

d Among those who reported ever having vaginal or anal sex.

e The range of GEMS scores is from 0 to 13 with a higher score representing more equitable gender norms views.

**Table 2.** Prevalence of Experiencing IPV Among South African AGYW Participating in HPTN 068 Baseline, March 2011-December 2012 (*n* = 2,533).

Characteristics	Ever experienced, total study population (*n* = 2,533)		Ever experienced, among those who reported ever being sexually active (vaginal or anal sex; *n* = 693)		Experienced in past 12 months, total study population (*n* = 2,533)		Experienced in past 12 months, among those reporting greater than one sexual partner in the past 12 months (*n* = 683)	
	Numerator/denominator	Prevalence (95% CI)	Numerator/denominator	Prevalence (95% CI)	Numerator/denominator	Prevalence (95% CI)	Numerator/denominator	Prevalence (95% CI)
Any violence (physical or sexual) by a partner (any IPV)	479/2,451	19.5 [18.0, 21.2]	256/684	37.4 [34.0, 41.2]				
Any physical violence by a partner	431/2,474	17.4 [16.0, 19.0]	239/689	34.7 [31.3, 38.4]	266/2,474	10.8 (9.6, 12.0)	159/681	23.4 [20.4, 26.8]
Partner slapped you or threw something at you that could hurt	291/2,485	11.7 [10.5, 13.1]	193/690	28.0 [24.8, 31.5]	179/2,484	7.2 (6.3, 8.3)	116/681	17.0 [14.4, 20.1]
Partner ever pushed or shoved	218/2,482	8.8 [7.7, 10.0]	116/690	16.8 [14.2, 19.9]	128/2,481	5.2 (4.4, 6.1)	81/681	11.9 [9.7, 14.6]
Partner ever hit you with his fist or with something else that could hurt you	78/2,482	3.1 [2.5, 3.9]	45/689	6.5 [4.9, 8.7]	53/2,482	2.1 (1.6, 2.8)	35/681	5.1 [3.7, 7.1]
Partner ever kicked you, dragged you, or beat you up	138/2,483	5.6 [4.7, 6.5]	77/689	11.2 [9.1, 13.8]	92/2,483	3.7 (3.0, 4.5)	61/681	9.0 [7.1, 11.4]
Partner ever choked or burned you on purpose	36/2,481	1.5 [1.1, 2.0]	22/689	3.2 [2.1, 4.8]	28/2,481	1.1 (0.8, 1.6)	20/681	2.9 [1.9, 4.5]
Partner ever threatened to use or used a gun, knife, or other weapon against you	37/2,484	1.5 [1.1, 2.1]	26/689	3.8 [2.6, 5.5]	25/2,484	1.0 (0.7, 1.5)	18/681	2.6 [1.7, 4.2]
Any sexual violence by a partner^a^	113^b^/2,490	4.5 [3.8, 5.4]	57/685	8.3 [6.5, 10.7]				
Ever physically forced to have sex when you did not want	76/2,498	3.0 [2.4, 3.8]	42/686	6.1 [4.6, 8.2]				
Ever had sex that you did not want because you were afraid of what the other person might do	66/2,503	2.6 [2.1, 3.4]	37/687	5.4 [3.9, 7.4]				
Both physical and sexual violence by a partner (both IPV)^a^	62/2,451	2.5 [2.0, 3.2]	39/684	5.7 [4.2, 7.7]				

*Note.* IPV = intimate partner violence; HPTN = HIV Prevention Trials Network; ACASI = Audio Computer-Assisted Self-Interview; AGYW = adolescent girls and young women.

a Question on sexual IPV experienced in the past 12 months could not be analyzed given ACASI construction.

b 56/113 girls said yes to sexual violence by a partner, but no to any vaginal or anal sex ever.

**Table 3.** Prevalence of Any Physical or Sexual IPV (Any IPV) Ever Among Those With Selected Characteristics, South African AGYW Participating in HPTN 068 Baseline, March 2011-December 2012—Total Study Population (*n* = 2,533).

	Among 17- to 20-year olds		Among 15- to 16-year olds		Among 13- to 14-year olds	
Characteristic (exposed status presented)	Number of any IPV/total *n* exposed status	Prevalence of any IPV ever among exposed status (95% CI)	Number of any IPV/total *n* exposed status	Prevalence of any IPV ever among exposed status (95% CI)	Number of any IPV/total *n* exposed status	Prevalence of any IPV ever among exposed status (95% CI)
Orphan (both/one parent dead)	75/207	36.2 [30.2, 43.4]	52/274	19.0 [14.9, 24.2]	20/191	10.5 [6.9, 15.9]
In past 12 months, worried about food for self or family	100/276	36.2 [31.0, 42.4]	83/359	23.1 [19.2, 27.9]	32/206	15.5 [11.3, 21.4]
In past 12 months, borrowed money from someone outside home	91/185	49.2 [42.5, 57.0]	55/234	23.5 [18.7, 29.6]	24/131	18.3 [12.8, 26.3]
Currently has savings for the future (cash, assets)	60/147	40.8 [33.6, 49.6]	53/260	20.4 [16.0, 25.9]	27/211	12.8 [9.0, 18.2]
Assets (AGYW with ≤13 durable goods in the home)	119/373	31.9 [27.5, 37.0]	100/547	18.3 [15.3, 21.8]	41/361	11.4 [8.5, 15.2]
Ever had vaginal sex	163/366	44.5 [39.7, 49.9]	72/254	28.4 [23.3, 34.5]	12/45	26.7 [16.4, 43.3]
Age at vaginal sexual debut^a^ (15 years or younger)^b^	47/87	54.0 [44.5, 65.6]	NC^c^	NC^c^	NC^c^	NC^c^
Ever had anal sex	30/52	57.7 [45.7, 72.8]	16/55	29.1 [19.3, 44.0]	3/8	37.5 [15.3, 91.7]
Number of lifetime sexual partners (two or more partners)^d^	101/203	50.0 [43.3, 57.1]	35/104	33.7 [25.7, 44.1]	6/11	54.6 [31.8, 93.6]
Number of sex partners in the past 12 months (one or more)^d^	159/348	45.7 [40.7, 51.2]	71/234	30.3 [25.0, 36.8]	9/40	22.5 [12.7, 40.0]
Unprotected sex with a partner in the past 3 months^d^	68/125	54.4 [46.3, 63.9]	26/71	36.6 [27.0, 49.7]	4/7	CU^e^
One or more of the three most recent sexual partners is/was a main partner/boyfriend^d^	123/274	44.9 [39.4, 51.2]	54/190	28.4 [22.7, 35.6]	10/31	32.3 [19.4, 53.7]
One or more of the three most recent sexual partners is/was a regular, casual sex partner^d^	58/117	49.6 [41.3, 59.5]	28/73	38.4 [28.7, 51.3]	2/8	25.0 [7.5, 83.0]
One or more of the three most recent sexual partners is/was a nonregular casual sex partner^d^	22/49	44.9 [32.9, 61.2]	11/38	29.0 [17.6, 47.6]	2/7	28.6 [8.9, 92.2]
One or more of the three most recent sexual partners is/was a sex work client^d^	7/11	63.6 [40.7, 99.5]	1/2	CU^e^	1/3	CU^e^
Currently lives with one or more (up to three) of the three most recent sexual partners^d^	28/53	52.8 [41.0, 68.1]	12/26	46.2 [30.5, 69.9]	1/5	CU^e^
Had transactional sex (sex because given in US$ or gifts)^d^	37/57	64.9 [53.6, 78.6]	13/34	38.2 [24.9, 58.6]	1/6	16.7 [2.8, 100.0]
HIV+	15/39	38.5 [25.9, 57.2]	5/24	20.8 [9.6, 45.4]	2/17	11.8 [3.2, 43.3]
HSV-2+	29/74	39.2 [29.5, 52.1]	7/39	18.0 [9.2, 35.1]	1/5	CU^e^
Older partner (≥5 years, sexual or nonsexual)	33/74	44.6 [34.6, 57.5]	21/66	31.8 [22.4, 45.3]	4/12	33.3 [15.0, 74.2]
GEMS score of 0–4^f^	149/387	38.5 [34.0, 43.7]	125/588	21.3 [18.2, 24.8]	48/425	11.3 [8.7, 14.7]
Ever pregnant	85/166	51.2 [44.1, 59.4]	21/50	42.0 [30.3, 58.2]	CU^e^	CU^e^
Currently using birth control	133/310	42.9 [37.7, 48.8]	73/254	28.7 [23.7, 34.9]	12/79	15.2 [9.0, 25.6]
Consumes any alcohol	46/81	56.8 [47.0, 68.7]	42/97	43.3 [34.5, 54.4]	11/45	24.4 [14.6, 40.9]
Ever used drugs	26/40	65.0 [51.8, 81.6]	12/47	25.5 [15.7, 41.6]	5/33	15.2 [6.8, 34.0]

*Note.* IPV = intimate partner violence; AGYW = adolescent girls and young women; HPTN = HIV Prevention Trials Network; HSV = herpes simplex virus; GEMS = gender equitable measurement scale.

a Vaginal sexual debut of ≤5 was considered unlikely and not included (*n* = 31).

b Among those who reported ever having vaginal sex.

c NC = not calculated as adolescent girls would not have a chance to experience later debut.

d Among those who reported ever having vaginal or anal sex.

e CU = numbers not large enough—estimate unstable or could not be calculated correctly.

f The range of GEMS scores is from 0 to 13 with a higher score representing more equitable gender norms views; median of 4.

**Table 4.** Baseline Associations of Selected Characteristics and Any Physical or Sexual IPV (Any IPV) Ever Among South African AGYW Participating in HPTN 068 Baseline, March 2011-December 2012—Total Study Population (*n* = 2,533).

	Among 17–20 year olds	Among 15–16 year olds	Among 13–14 year olds
Characteristics	Prevalence ratio (95% CI)	Prevalence ratio (95% CI)	Prevalence ratio (95% CI)
Orphan (both/one parent dead vs. both alive)	1.2 [1.0, 1.5]	1.1 [0.9, 1.5]	1.0 [0.6, 1.6]
In past 12 months, worried about food for self or family vs. did not worry	1.2 [1.0, 1.5]	1.6 [1.2, 2.0]	1.7 [1.1, 2.6]
In past 12 months, borrowed money from someone outside home vs. did not	1.9 [1.6, 2.4]	1.5 [1.1, 2.0]	2.0 [1.3, 3.1]
Currently has savings for the future (cash, assets) vs. currently does not	1.4 [1.1, 1.7]	1.2 [0.9, 1.6]	1.3 [0.8, 2.0]
Assets (AGYW with ≤13 durable goods in the home vs. not)	1.0 [0.8, 1.2]	1.1 [0.8, 1.4]	1.1 [0.7, 1.7]
Ever vaginal sex vs. never vaginal sex	2.5 [1.9, 3.3]	2.0 [1.5, 2.6]	2.7 [1.6, 4.6]
Age at vaginal sexual debut^a^ (15 years or younger vs. 16 years or older)^b^	1.3 [1.0, 1.7]	NC^c^	NC^c^
Ever anal sex vs. never anal sex	1.9 [1.5, 2.5]	1.7 [1.1, 2.6]	3.6 [1.4, 8.9]
Number of lifetime sexual partners (two or more partners vs. one)^d^	1.3 [1.0, 1.6]	1.3 [0.9, 1.9]	3.1 [1.3, 7.6]
Number of sex partners in the past 12 months (one or more vs. zero)^d^	1.1 [0.6, 2.1]	1.8 [0.6, 5.2]	0.5 [0.1, 1.4]
Unprotected sex with a partner in the past 3 months vs. no unprotected sex in past 3 months^d^	1.4 [1.1, 1.7]	1.4 [0.9, 2.0]	CU^e^
One or more of the three most recent sexual partners is/was a main partner/boyfriend vs. not^d^	1.0 [0.8, 1.3]	0.9 [0.6, 1.3]	2.4 [0.6, 9.7]
One or more of the three most recent sexual partners is/was a regular, casual sex partner vs. not^d^	1.2 [0.9, 1.5]	1.5 [1.0, 2.2]	1.0 [0.3, 3.5]
One or more of the three most recent sexual partners is/was a nonregular casual sex partner vs. not^d^	1.0 [0.7, 1.4]	1.0 [0.6, 1.7]	1.1 [0.3, 4.0]
One or more of the three most recent sexual partners is/was a sex work client vs. not^d^	1.4 [0.9, 2.3]	1.7 [0.4, 6.9]	1.3 [0.2, 7.0]
Currently lives with one or more (up to three) of the three most recent sexual partners vs. does not^d^	1.2 [0.9, 1.6]	1.7 [1.1, 2.7]	0.7 [0.1, 4.4]
Had transactional sex (sex because given US$ or gifts) vs. did not^d^	1.6 [1.3, 2.0]	1.4 [0.9, 2.2]	0.6 [0.1, 3.7]
HIV+ vs. HIV−	1.2 [0.8, 1.8]	1.2 [0.5, 2.6]	1.1 [0.3, 4.1]
HSV-2+ vs. HSV-2−	1.3 [0.9, 1.7]	1.0 [0.5, 2.0]	1.9 [0.3, 10.9]
Older partner (≥5 years, sexual or nonsexual)	1.1 [0.8, 1.4]	1.2 [0.8, 1.8]	1.4 [0.6, 3.3]
Gender equitable norms score of 0–4^f^	1.6 [1.3, 2.1]	1.6 [1.2, 2.2]	1.1 [0.7, 1.7]
Ever pregnant vs. never pregnant	2.0 [1.6, 2.5]	2.6 [1.8, 3.7]	CU^e^
Currently using birth control vs. not	1.9 [1.5, 2.4]	2.0 [1.6, 2.6]	1.5 [0.8, 2.6]
Consumes any alcohol vs. none	2.0 [1.6, 2.5]	2.9 [2.2, 3.8]	2.5 [1.4, 4.3]
Ever used drugs vs. never used drugs	2.2 [1.7, 2.8]	1.5 [0.9, 2.5]	1.4 [0.6, 3.3]

*Note.* IPV = intimate partner violence; AGYW = adolescent girls and young women; HPTN = HIV Prevention Trials Network; HSV = herpes simplex virus; GEMS = gender equitable measurement scale.

a Vaginal sexual debut of ≤5 was considered unlikely and not included (*n* = 31).

b Among those who reported ever having vaginal sex.

c NC = not calculated as adolescent girls would not have a chance to experience later debut.

d Among those who reported ever having vaginal or anal sex.

e CU = numbers not large enough—estimate unstable and could not be calculated, or the prevalence in Table 3 was not able to be calculated.

f The range of GEMS scores is from 0 to 13 with a higher score representing more equitable gender norms views; median of 4.
